# Seeding Aggregation Assays in Lewy Bodies Disorders: A Narrative State-of-the-Art Review

**DOI:** 10.3390/ijms251910783

**Published:** 2024-10-07

**Authors:** Anastasia Bougea

**Affiliations:** 1st Department of Neurology, “Aiginition” Hospital, School of Medicine, National and Kapodistrian University of Athens, 11528 Athens, Greece; abougea@med.uoa.gr; Tel.: +30-2107251315

**Keywords:** alpha-synuclein, Lewy body disease (LBD), real-time quaking-induced conversion (RT-QuIC), protein misfolding cyclic amplification PMCA, seed amplification assay (SAA), prion-like diseases

## Abstract

Multiple system atrophy and Lewy body diseases (LBDs) such as Parkinson’s disease, dementia with Lewy bodies, and Parkinson’s disease with dementia, known as synucleinopathies, are defined neuropathologically by the accumulation and deposition of aberrant protein aggregates, primarily in neuronal cells. Seeding aggregation assays (SAA) have significant potential as biomarkers for early diagnosis, monitoring disease progression, and evaluating treatment efficacy for these diseases. Real-time quaking-induced conversion (RT-QuIC) and Protein Misfolding Cyclic Amplification (PMCA) assays represent two ultrasensitive protein amplification techniques that were initially tested for the field of prion disorders. Although the fundamental idea behind the creation of these two methods is very similar, their technical differences resulted in different levels of diagnostic accuracy for the identification of prion proteins, making the RT-QuIC assay the most trustworthy and effective instrument for the detection of suspected cases of LBDs and prion-like diseases.

## 1. Introduction

Aggregated α-synuclein is crucial in the pathophysiology of synucleinopathies such as multiple system atrophy (MSA) and Lewy body disease (LBD) [1]. LBD is characterized by the presence of eosinophilic cytoplasmic inclusions, known as Lewy bodies (LBs) and Lewy neurites, whose basic component is alpha–synuclein (α-syn). It encompasses Parkinson’s disease (PD) (both sporadic and 5% genetic cases), dementia with Lewy bodies (DLB), and PD with dementia (PDD) [2]. The term “SWEDD” refers to scans without evidence for dopaminergic deficit, which is the absence of an imaging abnormality in patients who are clinically suspected of having PD [3]. There are two main MSA clinical classifications: MSA with major Parkinsonism and MSA with predominant cerebellar ataxia [4]. MSA is characterized neuropathologically by the presence of glial cellsynaptic inclusions (GCIs) in oligodendrocytes, which are also known as Papp-Lantz’s bodies. A set of neurologic illnesses known as pure autonomic failure (PAF), idiopathic REM sleep behavior disorder (iRBD), and perhaps idiopathic anosmia are defined by the formation of α-synaggregates, which are neuropathologically similar to LBD and MSA. The phrase “prodromal forms of synucleinopathies” refers to these illnesses, which are clinically and neuropathologically restricted types of synucleinopathies with a high probability of phenoconversion to PD, DLB, or MSA [5]. Even while LB syndromes exhibit distinct clinical distinctions, it can be challenging to differentiate between them in clinical practice due to a high rate of misdiagnosis [6,7].

Currently, the diagnosis of LBD is based on the observation of clinical symptoms, which typically appear when the neurodegeneration has progressed. In contrast, imaging methods and currently recognized diagnostic biomarkers have poor predictive values or are not feasible to use during the preclinical stages of the LBD [8]. It is therefore crucial to create better and more precise diagnostic instruments that could enable patients with LBD to receive an earlier diagnosis. The foundation of seeding aggregation assays (SAAs) is the idea that α-syn behaves like a prion, that is, misfolded, aggregated forms of the protein can cause otherwise normal α-syn to misfold and aggregate, which in turn encourages the formation of insoluble fibrils [9,10]. Cerebrospinal fluid (CSF) and brain homogenates (BH) are the perfect source of these aggregates because of their close proximity to the central nervous system (CNS); however, other biological liquids and peripheral tissues may also be promising.

This narrative state-of-the-art review aims to present the fundamentals of SAAs in various biological liquids and tissues, their crucial role in LBD diagnosis, their challenges/limitations, and recommendations for this exciting area of molecular research.

## 2. Basic Principles of Protein Amplification Methods

Originally developed for the field of prion disorders to detect PrPsc, the “Protein Misfolding Cyclic Amplification (PMCA)” and the “Real-Time Quaking-Induced Conversion (RT-QuIC)” are two ultrasensitive protein amplification methods for the identification of pathological protein aggregates [1]. Soto et al. developed PMCA in 2001 [1], while Atarashi et al. developed RT-QuIC in 2011 [11].

**Figure 1:**
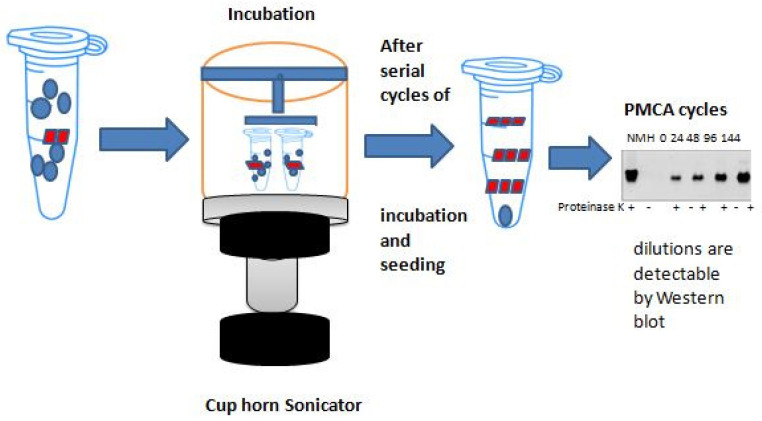
**Graphic presentation of PMCA method.** Small amounts of PrP^SC^ are amplified by PMCA to a measurable degree. PrPsc seeds from a sample are amplified cyclically via two phases (sonication and incubation) at the expense of excess PrP^c^. The big polymers break apart during the incubation stage to produce several smaller PrPs seeds for more prion replication.

These methods replicate the aggregation and misfolding of proteins observed in CJD in vivo and in vitro [12]. These methods’ basic concept is similar to that of a polymerase chain reaction (PCR) in that a template (protein aggregation) grows in a cyclic reaction at the expense of the substrate (protein monomer), increasing the number of template units [1] (Supplementary Materials) (Figure 1).

Despite using a similar strategy, there are several significant differences between the two methods. Whereas the substrate for RT-QuIC is a recombinant PrPc, the substrate for PMCA was obtained from extracts of hamster brains. Furthermore, in PMCA, sonication is used to encourage the monomeric substrate’s seeded aggregation, but in RT-QuIC, forceful intermittent shaking is used. The benefit of easily automating this intermittent shaking is that it can also be performed in fluorometers. PK digestion and Western blot (WB) analysis were used in PMCA at the conclusion of the process to identify and characterize the abnormal aggregates. However, in RT-QuIC, the WB analysis was substituted by a real-time recording of the thioflavin-T(ThT) dye’s fluorescence (Figure 1).

After binding to fibrils, the ThT fluorescence is stimulated at 450 nm and produces a strong signal at 480 nm. One of its limitations when compared to the WB analysis is that ThT fluorescence is only sensitive when there is a high concentration of cross-β sheets in the fibrillary aggregates. However, the ThT test requires less time when conducted in multi-well plates. These factors explain why, even though both approaches are highly sensitive and specific, their accuracy in identifying patients with sCJD varied [1]. As a result, the most reliable and efficient tool for early PrPsc identification in suspected cases of sCJD is the RT-QuIC test [12].

**Figure 2:**
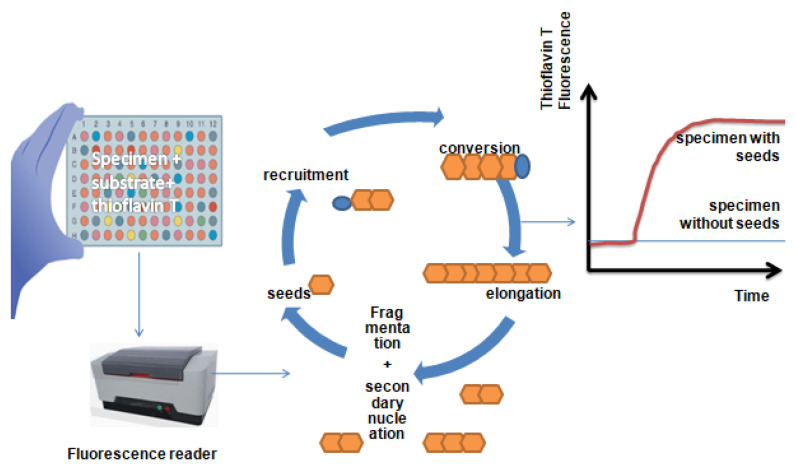
**Real-time quaking-induced conversion assay schematic**. Test specimens, solutions of substrate protein monomers, and an amyloid-sensitive dye (thioflavin T) are added to multi-well plates. The plates are placed in a temperature-controlled plate reader and shaken periodically to measure fluorescence. Amplification cycle: As monomers are drawn into the expanding fibril, seeds in the specimen bind to them and cause a conformational change. In addition to fragmentation, secondary nucleation can also result from the assembly and conversion of monomers on the sidewalls of fibrils, which can help create additional seeding surfaces. Readout for fluorescence: The fluorescence of the dye is increased when it binds to amyloid fibrils. When there are no pre-existing seeds in the specimen, spontaneous nucleation is necessary for the development of amyloid fibrils.

When PrPsc is present, the RT-QuIC assay facilitates, in vitro, the misfolding and aggregation process of PrPc, which occurs in vivo (Figure 2).

We need new seeding surfaces in order to achieve exponential growth kinetics. However, under assay-specific conditions, this process is significantly slower than the growth and fragmentation of pre-existing seeds. The LAG in responses that contain pre-existing seeds is indicative of the phase in which the seeds are growing at levels that are not observable. Once the accessible monomer is used up, the fluorescence reaches a plateau. Fluorescence can occasionally even drop after peaking; this phenomenon is probably caused by consolidation or redistribution of fibrils in the well in a way that influences the fluorescence measurement.

### α-syn-SAAs Protocols

Similar misfolded-protein amplification techniques have been applied in brain homogenates and CSF samples from patients diagnosed with synucleinopathies for the identification of misfolded α-syn due to the effectiveness of RT-QuIC technique for the detection of prion diseases and because α-syn seems to follow similar mechanisms of aggregation to the prion protein [1]. These tests include the recently published “α-syn-PMCA” assay, which is methodologically comparable to RT-QuIC and PMCA. They are collectively referred to as “seed amplification assays” (SAAs) [1,13,14]. Numerous studies to date have shown that α-syn seed amplification assays (α-syn-SAAs) performed on CSF may accurately and sensitively identify between individuals with PD and healthy controls (HC). Furthermore, even at the preclinical stage, such as in instances with isolated RBD, these assays demonstrated excellent sensitivity and specificity in identifying α-syn-SAAs [13]. According to the encouraging findings thus far, α-syn-SAAs may prove to be useful biomarkers for diagnosis in the field of synucleinopathies [1,13,14].

The first α-syn-RT-QuIC protocol was created by Fairfoul et al. [15]. Using human recombinant full-length α-synuclein as a substrate, Fairfoul et al.’s [15] approach involved incubating the samples at 30 °C in a phosphate buffer with a pH of 8.2. The regimen comprised twofold orbital shaking in the presence of zirconium/silica beads, with cycles of shaking (200 rpm) for one minute and resting for fourteen minutes. Every fifteen minutes, ThT fluorescence measurements were made. Following the protocol’s creation, Fairfoul et al. examined a preliminary panel of CSF samples from controls, mixed disease patients, and synucleinopathies with autopsy confirmation. Eleven of the twelve instances of pure DLB, both cases of PD, eleven of the seventeen cases of mixed DLB-Alzheimer’s disease, and two of the thirteen AD cases with incidental Lewy bodies had positive results; as a result, the sensitivity for pure DLB was 92%, and for mixed DLB-AD, it was 65%. The specificity was 100% because all cases of tauopathy, pure AD cases, and healthy controls obtained negative results.

In the validation stage, CSF samples from twenty participants with PD, fifteen controls, and three people with isolated RBD were examined by Fairfoul et al. [15]. A 95% sensitivity for PD and 100% specificity for separating PD from controls were obtained with the α-syn-RT-QuIC test [15]. The results from all three iRBD cases were also positive, meaning that this test might identify α-synucleinopathies in prodromal phases for the first time [4,15].

In 2017, Shahnawaz et al. [16] created an additional α-syn-PMCA protocol. Notably, in terms of methodology, this α-syn PMCA assay resembled RT-QuIC more than the original PMCA assay created by Soto et al. in 2001 [12]. Specifically, they used human recombinant full-length α-syn as a substrate rather than the substrate generally used in PMCA methods, which is produced from hamster brain extract [1,15]. Rather than utilizing sonication for the aggregation process and PK digestion followed by WB analysis for aggregate detection [1], they employed intermittent shaking, which involved cycles of 500 rpm shaking for one minute, 29 min of rest, and periodic ThT fluorescence measurements [16]. The reaction buffer included piperazine-N,N’-bis(ethanesulfonic acid) (PIPES) with a pH of 6.5 and 500 mM NaCl. The incubation temperature was set at 37 °C.

After being clinically diagnosed with synucleinopathies, CSF samples from 76 patients with PD, 10 patients with dementia-like symptoms (DLB), and 10 patients with MSA were tested using the α-syn-PMCA protocol. Control samples came from 14 patients with Alzheimer’s disease (AD), 17 patients with other neurodegenerative diseases, and 65 patients with other neurologic conditions but no neurodegenerative disorders. The assay demonstrated a total specificity of 94% for all control cases, with a sensitivity of 88% for PD, 100% for DLB, and 80% for MSA cases. The specificity increased to 96.9% for non-neurodegenerative control cases. Notably, two of the positive-scored samples in the non-neurodegenerative control group came from subjects who received a clinical PD diagnosis after one and four years, respectively. This suggests that α-syn-PMCA may be able to identify the disease even before symptoms appear [16]. Nine out of fourteen AD cases and fifteen out of eighteen non-synucleinopathy neurodegenerative disorders proved negative for the assay. At last, they discovered a possible association between α-syn-PMCA and the disease’s severity.

In 2018, Groveman et al. [17] created a third α-syn-RT-QuIC methodology. This research team employed a human recombinant α-syn containing the K23Q mutation as the substrate in this technique. Additionally, they used a phosphate buffer with a pH of 8.0, 170 mM NaCl, and an incubation temperature of 42 °C. The samples were subjected to double orbital shaking, with glass or silica beads present, in cycles of one minute of shaking (400 rpm) and one minute of rest. Every 45 min, ThT fluorescence measurements were made. The two other methods for α-syn-RT-QuIC and α-syn-PMCA required five to thirteen days to identify seeds, but this technique detected seeding activities more quickly—within one to two days [12]. The findings demonstrated a sensitivity of 92% for PD, 94% for DLB cases, and a total specificity of 100% for both controls and AD-cases.

BH is thought to be the best sample for detecting α-syn-aggregates [17]. Groveman et al. [18] obtained positive results in all samples and a higher seeding activity than in CSF samples when they performed RT-QuIC analysis on brain tissue samples from the frontal cortex in one PD and three DLB cases. According to previous reports [19,20,21], the RT-QuIC test in BH samples can discriminate DLB cases from controls with a sensitivity of 100% and specificities ranging from 83.3% to 100%. Additionally, a sensitivity of 90.9% was demonstrated for the identification of PD patients using BH samples [20].

CSF samples and the RT-QuIC and PMCA assays were used to investigate the α-syn-seeding activity in synucleinopathy cases thus far [21]. The α-syn-PMCA assay in CSF samples was able to distinguish between PD patients and healthy controls with 88–100% sensitivity rates and 89.9–100% specificity rates in previous studies [9,16,22,23,24,25]. Research utilizing CSF samples from PD patients in active treatment and non-synucleinopathy controls has demonstrated sensitivity and specificity rates of 75–100% and 80–100%, respectively [14,15,17,20,24,26,27,28,29,30,31,32,33,34]. Evidence utilizing autopsy-derived CSF samples to evaluate the RT-QuIC assay has shown that it can distinguish between PD cases and controls with 98–100% sensitivity and 100% specificity [15,35].

In three independent studies [5,9,16], the PMCA assay in CSF samples from DLB cases yielded positive results in 100%, 84.6%, and 50% of cases. RT-QuIC was able to identify α-syn-aggregates in CSF samples from 92 to 100% of postmortem DLB cases [15,30,35,36]; however, it identified 79–100% of living DLB cases [16,26,28,30].

The α-syn-PMCA assay demonstrated 80–100% sensitivity in CSF samples from MSA patients [9,16,22,23]. The research showed significant variation in the RT-QuIC assay’s positive rates for MSA cases. More specifically, for CSF samples from living MSA patients, five independent studies yielded sensitivities of 35, 6.45, 75, 12%, and 33%, respectively [28,29,30,32,34]. Only one of the three post-mortem MSA cases’ CSF samples from the two distinct trials tested positive for the QuIC assay [30,36].

The initial meta-analysis of α-syn-RT-QuIC’s diagnostic accuracy in synucleinopathies was carried out by Wang et al. [31]. In terms of identifying LBD patients from controls, they were able to achieve a sensitivity of 91% and a specificity of 95%. The control group was made up of patients with MSA, patients with other neurological disorders, and healthy individuals. The LB group includes PD, DLB, PAF, iRBD, and mixed instances of LBD.

In order to assess the diagnostic accuracy of α-syn-SAAs in distinguishing synucleinopathies from controls using CSF samples, Grossauer et al. [37] carried out a systematic review and meta-analysis in 2023. The use of α-syn-SAAs in the differentiation of synucleinopathies from non-synucleinopathies demonstrated a total sensitivity of 88% and a total specificity of 95%. Within the synucleinopathy group, patients with PD, DLB, MSA, prodromal synucleinopathies, and neuropathologically verified LBs mixed with other pathologies were included, while patients with other neurodegenerative and neurological disorders and healthy subjects comprised the non-synucleinopathy group. The sensitivity and specificity of α-syn-SAAs were 91% and 96% for the differentiation of synucleinopathies with LBs from non-synucleinopathies, respectively; patients with established PD or DLB yielded a higher sensitivity of 92%. When comparing patients with MSA to those with non-synucleinopathies, α-syn-SAAs achieved a total sensitivity and specificity of 57% and 96%, respectively. In contrast, the RT-QuIC method alone produced a much lower sensitivity of 30% when used to distinguish between MSA and non-synucleinopathies [21] (Table 1).

The detection of α-syn-seeding activities in bodily fluids or peripheral tissues other than CSF and BH is of major interest due to its potential use as a biomarker for α-synucleinopathies. A substantial number of publications conducted recently have used the alpha-syn-SAAs in biological fluids and peripheral tissue samples, including olfactory mucosa, gastrointestinal tract, skin, serum, submandibular gland, and saliva. (Table 2).

Using RT-QuIC assay in olfactory mucosa samples, De Luca et al. [39] compared 29 patients with the clinical diagnosis of tauopathy (18 subjects with clinical CBD and 12 subjects with clinical PSP) and 18 patients with the clinical diagnosis of synucleinopathy (18 subjects with clinical PD and 11 subjects with clinical MSA). In 56% of PD patients, 82% of MSA patients, 16% of CBD patients, and 16% of PSP patients, the RT-QuIC test result was positive.

Stefani et al. [40] employed the α-syn-RT-QuIC approach to identify α-syn aggregates in olfactory mucosa samples from 59 healthy persons, 41 patients with PD, and 63 patients with isolated RBD. A total of 10.2% of healthy people, 46.3% of PD patients, and 44.4% of patients with isolated RBD were positive for α-syn-RT-QuIC. The findings revealed that, in comparison to the healthy persons, the groups with separate RBD and PD had a sensitivity of 45.2% and a specificity of 89.8%. Additionally, a link between the α-syn-RT-QuIC values and a few clinical markers in the individuals with isolated RBD was found in this study. More precisely, for patients with isolated RBD and olfactory impairment, α-syn-RT-QuIC demonstrated a sensitivity of 73% and a specificity of 81%, indicating a substantial correlation between olfactory dysfunction and α-syn-RT-QuIC results (*p* < 0.01). Neither the control group nor the PD group showed this connection.

Perra et al. [41] conducted a study in which they employed the alpha-syn-RT-QuIC test on olfactory mucosa samples. For the control group in this study, 38 patients with neurodegenerative and non-neurodegenerative illnesses unrelated to α-syn were enrolled, while 43 patients with a clinical diagnosis of probable DLB, prodromal DLB, or mixed DLB/AD were also included. The research yielded an 86.4% accuracy rate, 81.4% sensitivity, and 92.1% specificity. Just three patients—one with AD, one with PSP, and one with psychosis—tested positive from the control group. When CSF samples from 32 non-neurodegenerative controls and 16 subjects with probable DLB and mixed DLB/AD were tested using RT-QuIC, the results showed a sensitivity and specificity of 100% and 90.6%, respectively, for differentiating between probable DLB/mixed DLB/AD and controls [25].

In 2021, Bargar et al. [35] collected OM samples from 11 controls, 20 possible MSA-P patients, 10 probable MSA-C patients, and 13 potential PD patients. Two distinct laboratories (USA-lab, ITA-lab) used the RT-QuiC test to evaluate the materials. For PD patients, both labs produced results with 69% sensitivity and for MSA-P patients with 90% sensitivity. Only one MSA-C patient had a positive RT-QuiC test result from USA-lab. The controls had a 100% specificity at ITA-lab and a 91% specificity at USA-lab. Between the two labs, a 96% inter-rated agreement of data was obtained.

Zheng et al. [51] conducted a meta-analysis to examine the diagnosis accuracy of different biospecimens using α-syn-SAAs. While CSF samples achieved a sensitivity and specificity of 91% (95% confidence interval 0.89–0.92) and 95% (95% CI 0.94–0.96), respectively, OM samples could distinguish PD from healthy controls or non-neurodegenerative neurological patients with a sensitivity and specificity of 51%and 91%, respectively.

Fenyi et al. [48] used the PMCA approach to find α-syn aggregates in gastrointestinal biopsies from the antrum, sigmoid colon, or rectum of 18 PD patients and 11 healthy controls. While just 9% of healthy people responded positively to PMCA, 55.5% of PD patients did. Additionally, it was demonstrated that, among all biopsy sites, the rectum had the lowest sensitivity of just 25% in the PD group. Zheng et al. [27] demonstrated in their meta-analysis that PD patients could be distinguished from healthy controls by using α-syn-SAAs in GI tract samples. This was achieved with a sensitivity of 44%and a specificity of 92%.

Manne et al. [42] used frozen skin tissues and FFPE (formalin-fixed paraffin-embedded) skin slices in their skin-RT-QuIC autopsy-verified PD patients and controls. The use of frozen skin tissues produced 96% sensitivity and specificity for discriminating between PD cases (n = 25) and controls (n = 25). In contrast, the use of FFPE skin tissues produced 75% sensitivity and 83% sensitivity in 12 PD patients and 12 controls. Furthermore, the identification of misfolded proteins in skin samples as a marker of longitudinal advancement may have wider translational significance as a result of this peripheral biomarker discovery investigation.

Wang et al. [43] examined autopsy abdominal skin samples from twenty PD-positive and four non-PD-positive cadavers using the RT-QuIC and PMCA techniques. While the PMCA method yielded results with an 83% sensitivity and a 100% specificity, the RT-QuIC method demonstrated a 95% sensitivity and 100% specificity. Examining autopsy scalp skin samples from these twenty PD-affected cadavers and ten non-neurodegenerative control cadavers, PMCA results revealed a sensitivity of 83% and specificity of 100%, respectively. A sensitivity of 100% and a specificity of 100% were attained in RT-QuIC, indicating that scalp skin samples are more sensitive than abdomen skin samples. Using autopsy abdominal skin samples from an additional 27 PD cadavers and 39 non-neurodegenerative controls, the RT-QuIC approach demonstrated a total sensitivity of 94% and a specificity of 98%. Tests for α-syn seeding activity on autopsy abdominal skin samples from seven out of seven LBD cadavers and two out of three MSA cadavers revealed a total sensitivity of 93% for all synucleinopathies. Skin samples from twelve out of seventeen AD cadavers, all five CBD cases, and eight PSP cases showed negative results for patients with other neurodegenerative disorders. Thus, for skin samples from all PSP, CBD, AD, and non-neurodegenerative cases, the specificity of RT-QuIC was 93%. When the PMCA technique was used to analyze a smaller subset of these samples—32 cadavers with synucleinopathies and 18 cadavers without—it was shown that the technique could distinguish between synucleinopathies and non-synucleinopathies with an 82% sensitivity and a 96% specificity.

Wang et al. [43] also examined skin samples obtained from leg and posterior cervical biopsies of twenty people with PD and twenty non-PD individuals who are still living. The sensitivity and specificity of the RT-QuIC analysis were 100% and 95%, respectively. The PMCA assay demonstrated 80% sensitivity and 90% specificity when evaluated on 10 PD patients and 10 living controls.

Another investigation, carried out in 2021 by Donadio et al. [44] examined the RT-QuIC assay in skin biopsy samples taken from the C7, thigh, and leg as well as CSF samples from patients with PD, LBD, MSA, PAF, cases of non-synucleinopathy, and controls. Skin biopsy RT-QuIC testing yielded results with specificities of 83% for controls and 70% for non-neurodegenerative disorders, sensitivities of 100% for PD, 60% for MSA, 75% for LBD, and 67% for PAF. While controls and non-neurodegenerative cases obtained a specificity of 100%, CSF-RT-QuIC reached sensitivities of 100% for PD, 50% for MSA, 100% for LBD, and 50% for PAF [43].

To test the diagnostic value of RT-QuIC assay in patients with LBD, Mammana et al. [45] collected skin samples from the cervical region and the thigh in a group of 49 postmortem individuals and from the leg, thigh, or cervical region in a group of 69 living individuals. One DLB case, one PD subject, seven subjects with incidental LB pathology, and forty non-LBD subjects were included in the postmortem group. In contrast to the iLBD cases, which showed a sensitivity of 85.7% and 66.7% for cervical and thigh skin samples, respectively, the PD and DLB cases both tested positive with RT-QuIC. Of the forty remaining non-LBD individuals, all skin samples except for one cervical sample had a negative response in this assay, revealing a specificity for thigh skin samples of 100%. The postmortem group achieved a sensitivity of 88.9% in differentiating synucleinopathies from non-synucleinopathies. There were fifteen DLB patients, thirteen PD patients, and forty-one healthy people in the group of living people. All DLB cases, ten out of thirteen PD cases, and two out of forty-one healthy individuals had positive skin samples. The test’s overall results for sensitivity and specificity in identifying DLB/PD patients from controls were 89.3% and 95.1%, respectively [45].

The RT-QuIC assay was used to evaluate the CSF samples from 79 patients in both groups, enabling a comparison of the CSF and skin samples from these patients. All thirty non-LBD cases, one out of four iLBD cases, and zero out of one PD-DLB case tested negative with RT-QuIC from the postmortem group, while none of the twenty-seven controls and all eighteen PD and DLB CSF samples from the live patients were positive. Between skin and CSF samples, the results were 96.2% concordant [45].

In 2021, Kuzkina et al. [46] gathered several skin biopsy samples from 30 healthy persons and 34 patients with PD in order to evaluate the skin RT-QuIC’s diagnostic usefulness in PD. A total of 81 biopsies from healthy participants and 117 samples from PD patients were taken from the neck C7, thigh, lower back Th10, and lower leg. The results of the numerous skin biopsies showed an 88.9% diagnosis accuracy, with the best separation between PD patients and controls observed at the C7 biopsy location. Interestingly, this study found a correlation between a higher α-syn seeding activity and the presence of clinical symptoms such as constipation, RBD, and cognitive impairment, as well as a longer duration and more advanced stage of the disease.

In a different investigation, which was carried out by Kuzkina et al. [47] in 2023, 38 iRBD patients, 39 PD patients, and 23 controls were examined for seeding activity using the dermal RT-QuIC assay. Skin samples were obtained from the proximal leg, TH10, and/or C7. The findings demonstrated a specificity of 87% in differentiating between patients and healthy controls, and a sensitivity of 97.4% for iRBD patients and 87.2% for PD patients. Th10 demonstrated the highest specificity of 100% with 21 controls when comparing the various biopsy locations.

According to Zheng et al. [51] meta-analysis, α-syn-SAAs in skin samples have 91% sensitivity and 92% specificity for differentiating between PD patients and healthy or non-neurodegenerative neurological controls.

Manne et al. [52] used the α-syn RT-QuIC assay to evaluate submandibular gland samples from autopsied people with PD, incidental LBD (n = 3), and controls (n = 16). The sensitivity for both groups was 100%agreement for higher levels of pathological α-syn seeding activity. Prodromal PD in submandibular gland tissues may be identified using the α-syn RT-QuIC assay, as evidenced by the discovery of seeding activity from incidental LBD cases harboring immunohistochemically undetectable pathogenic α-syn.

In a further study, submandibular gland tissues and CSF samples from PD patients and healthy controls were also subjected to α-syn-SAA testing by Chahine et al. [10]. CSF samples had a 92.6% sensitivity and a 90.5% specificity for the diagnosis of PD, whereas submandibular gland samples had a 73.2% sensitivity and a 78.6% specificity. Of the 38 individuals with PD, 65.8% showed improvement on the CSF RT-QuIC and SMG RT-QuIC.

According to a recent meta-analysis, PD patients could be distinguished from healthy controls and non-neurodegenerative neurological patients using α-syn-SAAs in submandibular gland samples with a sensitivity of 80%and a specificity of 87%. The invasiveness of the submandibular gland biopsy process renders these samples less desirable for clinical application, despite the encouraging outcomes of these research [4].

Salivary RT-QuICtest was used by Luan et al. [49] to investigate PD, MSA patients, and non-neurodegenerative controls. Salivary RT-QuIC identified PD patients with a sensitivity of 76% and a specificity of 94.4%, whereas a sensitivity of 61.1% was noted for MSA patients. In particular, patients with PD had a notably faster LAG phase in the RT-QuIC assay than patients with MSA. This finding may have practical applications in differentiating between PD and MSA.

Zheng et al. meta-analysis [51] revealed that saliva samples from PD patients had a sensitivity of 79% when α-syn-SAAs were used, whereas saliva samples from neurological or non-neurodegenerative healthy controls had a specificity of 88%.

In 2023, Okuzumi et al. [50] tested the utilization of a modified immunoprecipitation-based (IP) RT. QuIC assay to identify α-syn-SAAs in serum samples of patients with synucleinopathies. This was performed by conducting a series of cohorts. A total of two hundred twenty-one PD patients, thirty-nine MSA patients, ten DLB patients, nine RBD patients, thirty PSP patients, twenty-five AD patients, seventeen Parkin-linked PD patients, and one hundred twenty-eight non-neurodegenerative controls had their serum examined in the first cohort.

A total of 90% of DLB patients,44% of MSA patients, 90% PD patients, and 40% of RBD patients had positive results from the IP/RT-QuIC assay. Within the non-synucleinopathy group, the IP/RT-QuIC assay revealed that 117 controls, 21 AD patients, 29 PSP patients, and all Parkin-linked PD patients tested negative. Four of the six MSA patients, none of the nine non-neurodegenerative controls, and all thirty-four PD patients had favorable results from the second internal cohort. In the third external cohort, the IP/RT-QuIC assay yielded negative results in all six PSP patients and nineteen out of twenty non-neurodegenerative controls, but positive results in fifteen out of twenty PD patients and eight out of fifteen MSA patients.

In 2021, Bargar et al. [53] tested multiple samples from the brain, CSF, and scalp skin of one neuropathologically confirmed PD case and one AD case as control. They also tested multiple samples from the sigmoid colon, submandibular gland, scalp skin, and CSF of another neuropathologically confirmed PD case and a non-neurological control case. All tests were conducted using parallel RT-QuIC reactions. While no seeding activity was found in any of the samples from the two controls, the RT-QuIC assay found seeding activity in all of the samples from the two PD cases. This suggests that peripheral tissue samples may perform similarly to CSF samples when it comes to distinguishing between synucleinopathies and controls.

## 3. SAAs in Genetic PD

α-syn-SAAs have previously been used in a number of studies to assess genetic PD patients or carriers of mutations linked to PD or DLB [14,26,27,54]. Garrido et al. [27] examined CSF samples from 15 LRRK2(p.G2019S)-PD patients, 10 idiopathic PD patients, 16 LRRK2(p.G2019S) NMCs, and 10 healthy persons using the RT-QuIC assay. Patients with iPD and LRRK2-PD were detected by RT-QuIC with 90% sensitivity and 40% sensitivity, respectively. The specificity for healthy controls was 80%, while 18.8% of LRRK2 NMCs showed positive RT-QuIC findings.

The CSF RT-QuIC test was used in 236 PD patients with sporadic PD, PD with GBA, LRRK2, parkin, PINK-1, or DJ1 mutations; 49 DLB patients with either sporadic DLB or DLB with GBA mutation; 14 NMCs with recessive-associated PD gene mutations; or 26 healthy individuals by Brockmann et al. [26]. RT-QuIC shown a sensitivity of 91% for sporadic PD, 86.8% for GBA-PD, 78% for LRRK2-PD, and 50% for recessive PD in patients with PD. The results showed that NMCs and healthy controls had a specificity of 86% and 92%, respectively, while sporadic DLB patients had a sensitivity of 79% and GBA-DLB patients a sensitivity of 100%.

Brain tissues from Substantia nigra (SN), anterior cingulate (AC) gyrus, and ventricular CSF postmortem samples of PD subjects with LRRK2 mutation with and without LB pathology were tested by Garrido et al. [54]. All three of the LRRK2-PD cases with neuropathologically confirmed LB pathology had positive results in both brain and CSF samples; in contrast, three of the five cases with LRRK2-PD without neuropathologically confirmed LB pathology had positive results in CSF samples but all five had negative results in brain samples from both regions. The sensitivity of seven controls with LB pathology but no LRRK2 mutation was 100% for BH samples and 83% for CSF samples.

The last group, which comprised five non-LB pathology controls, had 100% specificity for both CSF samples and brain samples from SN, but only 60% specificity for brain samples from AC gyrus. In CSF samples, underlying LB pathology was detected with an overall sensitivity of 88.9% and a specificity of 100%; in contrast, SN samples had an overall sensitivity and specificity of 100% for LB pathology identification [54].

In the aforementioned work, Siderowf et al. [14] examined 310 NMCs with GBA or LRRK2 mutations and CSF samples from 545 PD patients. Among the patients with PD were 373 individuals with sporadic PD and 49 patients with genetic PD, who carried the GBA N370S mutation or the LRRK2 G2019S variant, respectively. The genetic profile revealed that patients with PD who had the GBA mutation had the highest sensitivity of 95.9%, followed by patients with sporadic PD (93.3% sensitivity), and patients with LRRK2 PD (67.5% sensitivity), who had a relatively low sensitivity. Among the clinical traits of PD patients, hyposmia was the most important factor in a favorable outcome. A total of 65.3% of PD patients who carried the LRRK2 gene but did not exhibit hyposmia had negative results; also, among LRRK2 PD cases, female gender was more likely than male gender to have an α-syn-SAA negative result. A total of 8.8% of LRRK2 carriers and 7.3% of GBA carriers who did not exhibit any symptoms of their gene mutations tested positive for α-syn-SAAs.

## 4. SAAs in MSA Variants

In order to determine whether there is a difference in the diagnostic accuracy of α-syn-SAAs between MSA-C and MSA-P patients, some research recorded the exact MSA variation of each MSA patient. According to Van Rumund et al. [28], the CSF RT-QuIC sensitivity for MSA-C (n = 4) was 0%, while the sensitivity for MSA-P (n = 13) was 46%. In 2021, Bargar et al. [35] conducted an interlaboratory investigation that yielded positive results for OM RT-QuIC in eighteen out of In total, twenty MSA-P patients by both laboratories and in just one out of ten MSA-C patients by one laboratory. These findings demonstrated that, in the RT-QuIC test, MSA-P and MSA-C produced opposing effects [35]. In these experiments [27,42], MSA-C patients did not generate seeding activity with RT-QuIC; however, this trend was not replicated in the three subsequent studies. Poggiolini et al. [29] used the RT-QuIC assay to examine CSF samples from nine patients with the MSA-C variation and fifteen patients with the MSA-P variant. A total of 100% of the MSA-C cases and 57% of the MSA-P cases had positive results. Donadio et al. [44] performed another study that produced skin-RT-QuIC sensitivity values of 100% for MSA-C (1 case) and 50% for MSA-P (4 cases). Okuzumi et al. [50] used the IP/RT-QuIC assay to test serum MSA samples with 62% sensitivity for the MSA-C variant (*n* = 13) and 65% sensitivity for the MSA-P variant (*n*= 26).

## 5. SAAs in Prodromal LBD

A sensitivity of 92.9% was observed by Rossi et al. [30] while applying the CSF RT-QuIC test for PAF patients (n= 28). Two of the three PAF patients’ skin samples and one of the two PAF patients’ CSF samples from a different research of Donadio et al. [44] had favorable outcomes with the RT-QuIC assay.

Singer et al. [23] analyzed CSF samples from PAF patients in 2021 and found that 30 out of 32 instances had reactive PMCA assays, proving that PAF is a synucleinopathy. In order to ascertain if these individuals will phenoconvert to PD, LDB, or MSA in the future, they were monitored prospectively. Of the thirty-two PAF patients who were originally included in the trial, five switched to MSA, two to DLB, and two to PD, all of whom had positive PMCA results at recruitment. Surprisingly all five of the PAF patients that phenoconverted to MSA eventually fell within the defined range of highest ThT fluorescence (150–2000 AU) in the initial PMCA assay [23].

Approximately 80% of patients with iRBD will eventually acquire a synucleinopathy, such as PD, DLB, or MSA, according to a number of long-term cohort studies [4,55]. One of the most prevalent non-motor signs of synucleinopathies is RBD. To be more precise, RBD is one of the four primary clinical symptoms used to diagnose DLB, and its absence is regarded as a warning sign for the diagnosis of PD five years into the illness. RBD may not yet be included in the clinical criteria for the diagnosis of MSA, although several studies have demonstrated that it is helpful in this regard [4].

RBD is thought to be a prodromal phase of synucleinopathies and a highly specific biomarker for a future synucleinopathy because it typically appears prior to the beginning of motor and other non-motor symptoms [4,55]. Kluge et al. [56] show that it is possible to identify and magnify pathogenic α-syn conformers in peripheral blood in both iRBD-positive and -negative patients up to 10 years prior to the clinical diagnosis of PD. Notably, the blood-based α-syn SAA assay may be promising biomarker in prodromal PD diagnosis.

Three iRBD patients’ CSF samples were examined by Fairfoul et al. [14] using RT-QuIC, and each of the three instances produced positive results. Using CSF RT-QuIC, 18 iRBD patients showed 100% sensitivity in another study conducted by Rossi et al. [30]. Iranzo et al. [38] analyzed CSF samples from 40 healthy people and 52iRBD cases; the results showed that the RT-QuIC assay has 90% sensitivity and 90% specificity. Poggiolini et al. [29] study showed a significantly lower sensitivity of 64% in identifying individuals with iRBD (n = 45). Concha-Marambio et al. [25] evaluated the application of the CSF PMCA assay in iRBD (n = 29) participants, and the results showed high sensitivity of 93.1%.

Several attempts have used α-syn-SAAs to analyze peripheral tissue samples in iRBD participants in addition to CSF samples. Using the OM RT-QuIC assay, Stefani et al. [40] evaluated OM samples from 63 iRBD participants and found a sensitivity of 44.4% for iRBD. Kuzkina et al. [47] analyzed skin samples from 38 iRBD patients in 2023 using the RT-QuIC technique, and the results showed a sensitivity of 97.4%. When IP/RT-QuiC assay was used in a study by Okuzumi et al. [50] to test serum samples of nine iRBD patients, the results showed that 44% of the patients had positive results.

Rossi et al. [30] utilized the CSF RT-QuIC assay to evaluate patients with mild cognitive impairment (MCI) resulting from likely LBD (n = 81), AD (n = 120) or unknown etiology (n = 30), and a control group (no = 58). The RT-QuIC test has a sensitivity of 95.1% for MCI-LBD and specificities of 86.7% for MCI-AD, 93.3% for MCI with an unknown etiology, and 96.6% for controls. Siderowf et al. [14] evaluated 51 prodromal patients; of these, 86.2% had CSF samples that were positive for the RT-QuIC assay. Patients without a PD diagnosis but exhibiting prodromal symptoms, such as hyposmia (n = 18) or RBD (n = 33), were included in the group of prodromal patients. Positive α-syn-SAA results were found in 16 out of 18 hyposmia participants and 28 out of 33 RBD participants; RBD cases with considerable olfactory loss were more likely to have positive results.

Perra et al. [41] presented early data suggesting that evaluating both the CSF and the olfactory mucosa of patients suffering from mixed and DLB together could potentially increase the concordance with clinical diagnosis to 100%.

The meta-analysis conducted by Grossauer et al. [37] demonstrated that the use of α-syn-SAAs in CSF samples could distinguish patients with prodromal synucleinopathies, such as RBD, PAF, MCI-LB, and NMCs of genetic mutations known to cause synucleinopathies, from cases of non-synucleinopathy with a sensitivity of 74% and a specificity of 93%.

## 6. SAAs in SWEDDs

Concha-Marambio et al. [25] analyzed CSF samples from 20 SWEDDs in 2021 using the PMCA assay, and 80% of the cases had negative results. It is interesting to note that two of the four SWEDDs with positive results had a significant increase in dopaminergic degeneration as detected by DaTscan at 42 and 46 months, respectively. This suggests that the individuals were not false positives but indeed cases of synucleinopathy. A different CSF-RT-QuIC assay study reported that the specificities for SWEDDs and healthy controls were 90.7% and 96.3%, respectively [14]. This indicates that the SWEDD group had a greater percentage of positive outcomes than the healthy control group.

## 7. Distinguishing between Synucleinopathies

Between MSA and PD, there is a variance in the neuroanatomical distribution, biochemical composition, and morphological characteristics of α-syn-aggregates; this variety appears to be caused by different α-syn strains [18].

Groveman et al. [17] shown in their study that the ThT amplitudes of the aggregation products with RT-QuIC of PD and DLB patients differed. According to a number of investigations [5,22,23] the maximal fluorescence signal of the α-syn fibrils formed by CSF samples from LBD patients, identified with α-syn-PMCA, was much higher than CSF samples from MSA patients.

More precisely, Singer et al. [23] found that individuals with MSA could be distinguished from PD/DLB patients with 100% and 93% sensitivity and specificity, respectively, within the range of 1.615 AU to 2.605 AU of the maximal ThT fluorescence cutoff value. Additionally, Shahnawaz et al. [22] demonstrated that PD patients’ ThT threshold values varied from 2.000AU to 8.000AU. The range of maximum ThT fluorescence cutoff values for MSA with PMCA was 150–2000 AU, after accounting for the cutoff values (80–491 AU) for discriminating between MSA and controls and the variability of these values in each disease. ThT amplitudes between MSA and LBD varied, according to some research that used the RT-QuiC test [29,50].

However, these variations did not appear in every study using the RT-QuIC test. For instance, Luan et al. [49] used RT-QuIC to detect salivary samples from individuals with PD and MSA and found no statistically significant variations in the intensity of ThT fluorescence of α-syn aggregates.

The aggregation kinetics of PMCA demonstrated a further distinction between MSA and LBD; in MSA samples, aggregate formation and polymerization happened significantly earlier than in LBD samples [5,23]. According to De Luca et al. [39], there was a notable difference in the resistance of the seeded RT-QuIC products from MSA samples to PK digestion when compared to the seeded products from PD samples.

With the goal of exploring the morphological features of the α-syn fibrils generated by α-syn-SAAs in different synucleinopathies, some studies also performed a transmission electron microscopy (TEM) analysis [39,49,54]. De Luca et al. [39] showed that there were significant differences in the morphological properties of the fibrils produced by the RT-QuIC assay between PD and MSA patients, despite Luan et al. [49] finding no statistically significant differences in the diameter of α-syn fibrils derived from salivary RT-QuIC between MSA and PD patients. Additionally, Okuzumi et al. [50] revealed significant differences between MSA, LBD, and RBD in the main morphologies and fibril widths formed from α-syn seeds with RT-QuIC. Other methods, including FTIR, cryon-electron tomography, and circular dichroism, were also used to demonstrate structural variations in the MSA and PD α-syn fibrils generated by PMCA [22], but more research is warranted.

Using aSyn-SAAs in CSF samples, Zheng et al.‘s meta-analysis [51] reported a sensitivity of 91% and a specificity of 50% for differentiating between PD and MSA. Skin samples produced 92% sensitivity and 22% specificityfor the use of α-syn-SAAs in the distinction of PD versus MSA patients, whereas OM samples produced 55% sensitivity and 50% specificity.

Several studies have proven that the PMCA and RT-QuIC assays can both identify LBD patients with high sensitivity and specificity. However, the PMCA assay has been presented to be more accurate for detecting MSA patients and differentiating between MSA and LBD [5,57]. As previously stated, the meta-analysis conducted by Grossauer et al. [37] revealed that the use of only the RT-QuIC method for MSA resulted in a much lower sensitivity of 30%, whereas the analysis of both PMCA and RT-QuIC assay yielded a total sensitivity of 57% for MSA patients. These findings imply that PMCA may be more effective than RT-QuIC assay in identifying MSA aggregates, along with the considerable variability of RT-QuIC results in MSA patients [57]. However, given these assays require laboratories with high experience and tight standardization to deliver precise outcomes in these disorders, these results could possibly be the result of technical optimization or staff performance concerns.

## 8. Challenges and Limitations

One of the key advantages of SAAs is their ability to detect pathological α-syn aggregates at very early stages of disease, even before the onset of clinical symptoms [56]. This has significant implications for early diagnosis and intervention, as current diagnostic methods often rely on the presence of motor or cognitive symptoms which may not manifest until the disease has already progressed significantly. By detecting α-syn aggregates early on, SAAs can help identify individuals at risk for developing LBD and initiate treatment interventions sooner.

Moreover, SAAs offer a unique opportunity to monitor disease progression over time. By measuring the accumulation and spread of pathological α-syn aggregates in the brain, researchers can track the development of LB pathology and assess the effectiveness of potential therapeutic interventions. This is particularly important in the context of clinical trials for new drugs targeting α-syn aggregation, as SAAs can provide valuable insights into the mechanisms of action and potential efficacy of these treatments.

In addition, SAAs have the potential to serve as objective biomarkers for LBD, complementing existing clinical assessments and imaging modalities. Biomarkers play a critical role in disease diagnosis, prognosis, and treatment monitoring, and there is currently a lack of reliable biomarkers for LBD. SAAs offer a promising avenue for the development of biomarkers that are specific to alpha-synuclein pathology, which is a hallmark feature of these diseases. In comparison to total α-syn measures, the α-syn SAA in CSF, a central tissue, demonstrated superior sensitivity and specificity (Youden Index = 83.1%) than the submandibular gland, a peripheral tissue proximal to the CNS [10]. Additionally, within-subject connections between central and peripheral α-syn measures were observed. The CNS may be the main reference for diagnosis.

Despite their promise, SAAs also face several challenges and limitations that need to be addressed. One of the main challenges is the variability in assay protocols and experimental conditions, which can lead to inconsistencies in results and hinder the reproducibility of findings across different studies [58]. The standardization of assay procedures and validation of results in large cohorts of patients will be critical for the widespread adoption of SAAs in clinical practice [38]. While some CSF investigations found no correlation [47], others found weak correlations between a short LAG phase and greater Imax with longer disease duration and higher MDS-UPDRS-III. A plausible rationale for the incongruous outcomes, which presently restrict the “quantitative” usefulness of α-syn-AA for CSF, is the significant variations linked to the many tests. This might be caused by variations in reagents or techniques, the comparatively small number of samples examined, or the intrinsic variability of the aggregation process itself. The extremely low biomarker concentrations in the CSF milieu are an essential part of the SAA reaction that have a direct impact on the reaction’s kinetic parameters. It has been demonstrated that CSF lipoproteins suppress the CSF SAA response in this way [59].

Moreover, the development of SAAs for use in human samples presents a major logistical challenge. Current assays are primarily performed in cell culture models or animal models, which may not fully capture the complexity of α-syn aggregation in the human brain. Translating these assays to human samples, such as CSF or brain tissue, will require careful optimization and validation to ensure their reliability and accuracy as biomarkers for LBD.

Another limitation of SAAs is their specificity for α-syn pathology, which may limit their utility in diagnosing other neurodegenerative diseases that share similar protein aggregation features. Creating assays that are more sensitive for identifying protein aggregates is another difficulty. The sensitivity and specificity of existing techniques, like fluorescence microscopy and biochemical approaches, are limited. Mass spectrometry and super-resolution microscopy are two examples of new imaging techniques that could offer more precise and thorough information regarding the structure of protein aggregates [60]. An alternate method that could help ensure a trustworthy measurement of the seed concentration is the end-point dilution. By calculating the number of samples with sufficient seeding activity to produce positive results in 50% of technical replicate reactions, this method compare the percentage of positive technical replicate reactions at each dilution, the biospecimen is tested in serial dilutions; however, few studies are conducted so far [35,61,62]. The RT-QuIC kinetic parameters and the number of positive replicates were considerably impacted by adding detergents and a blood contamination level of less than 0.01 percent among the preanalytical factors that were examined. Th reproducibility of the results was enhanced by increasing the number of duplicates. In repeatedly diluted CSF samples, the number of positive duplicates made it easier to distinguish between samples with high and low seeding activity. In multiple experiment settings, the most dependable kinetic measure was the time to threshold (LAG) [63]. While α-syn is a key player in Lewy body disorders, other proteins such as tau and β-amyloid also contribute to neurodegeneration in diseases like AD. Future research efforts should focus on developing seeding aggregation assays that can detect a broader range of pathological protein aggregates, enabling more comprehensive and accurate diagnosis of neurodegenerative diseases.

## 9. Future Directions

There are various prospective approaches for seeding aggregation experiments that show promise for improving research in this subject as our understanding of the role of α-synaggregation in LB diseases continues to develop. Developing more accurate and focused tests to identify and measure α-syn aggregates in biological samples like blood or CSF is one of the main areas of study. It will be essential to create animal models that accurately reflect the course of LB diseases in order to evaluate the effectiveness of prospective treatments. The sensitivity and specificity of the current techniques for measuring α-synaggregates, such as PMCA and enzyme-linked immunosorbent assays (ELISAs), are limited, making it difficult to diagnose LBD or track the course of the disease. Given the differences among each patient and their different stages of the disease, a comprehensive diagnosis based on multiple testing methods may be a more reliable approach. We may be able to create more accurate biomarkers for early illness detection and tracking by enhancing our capacity to identify and measure α-synaggregates using seeded aggregation tests.

Exploring new approaches to targetα-synaggregates and stop their spread in the brain is a significant future consequence of research on seeded aggregation tests. Although the majority of LBD current therapy approaches concentrate on managing symptoms, there is rising interest in creating disease-modifying medications that can stop or reverse the growth of α-synaggregation. Potential medication candidates that target particular stages in the aggregation pathway, such as nucleation, fibril elongation, or fragmentation, can be screened using seeding aggregation experiments. We may be able to create novel treatments that halt the advancement of LBD and enhance patient outcomes by finding tiny compounds or biologics that can prevent the development or spread of α-synaggregates.

Seeding aggregation tests are useful not only for drug development but also for examining the role of genetic and environmental variables in α-synaggregation and illness etiology. Numerous risk factors for LB problems, such as chemicals, head trauma, and genetic abnormalities, have been identified by epidemiological research. By shedding light on the ways in which these variables affect alpha-synuclein aggregation and neurodegeneration, seeding aggregation tests can shed light on the causes of disease and suggest possible lines of defense. Through the integration of several factors that contribute to the initiation and progression of LBD, researchers can create more comprehensive models by integrating data from seeded aggregation experiments, including genetic, environmental, and molecular data.

## 10. Conclusions

SAAs have revolutionized the field of α-syn research and have become indispensable tools for studying LBD. These assays offer a sensitive and reliable method for detecting and amplifying alpha-synuclein aggregates, making them well-suited for investigating the early stages of disease progression, disease mechanisms, and potential treatments. When it comes to reliable and efficient methods for detecting α-synaggregates, two very sensitive protein amplification techniques that were first investigated in the realm of prion disorders are the RT-QuIC and PMCA assays. Researchers can learn more about the molecular processes behind protein aggregation and neurodegeneration in LBD by honing and expanding these assays. This will eventually lead to the creation of more precise diagnostic instruments and focused therapeutic interventions. The development of new therapeutic targets to impede the propagation of aggregation, the investigation of the influence of environmental and genetic factors on disease pathogenesis, and the enhancement of sensitivity and specificity for the detection of α-synaggregates are the future directions for SAAs. We can become a step closer to solving the riddles of LBD and creating novel approaches to enhance patient outcomes by utilizing the potential of SAAs in conjunction with other molecular and cellular methodologies. Going forward, further refinements of SAAs and their application in larger cohort studies will likely provide new insights into the pathophysiology of LBD and facilitate the development of novel therapeutic strategies.

## Figures and Tables

**Table 1:** Studies on α-syn-SAAs using CSF and BH samples.

Author, Publication Year	Assay	Sample	Autopsy	Disease	Number of Samples (Cases/Controls)	Main Outcomes
Fairfoul et al., 2016 [15]	RT-QuIC	CSF	YES	DLB DLB/AD AD/iLBD PD PSP CBD	12/20 17/20 13/20 2/20 2 3	AD and control CSF: 100% specificity DLB and PD CSF CSF: 92% and 95% sensitivities, respectively. Negative results for PSP and CBD.
			ΝO	PD iRBD	20/15 3/15	
Shahnawaz et al., 2017 [16]	PMCA	CSF	NO	PD DLB MSA	76/97 10/97 10/97	Achieved 88.5% sensitivity and 96.9%. specificity. The degree of clinical symptoms of PD was associated with the α-syn-PMCA results for various patients.
Sano et al., 2017 [19]	RT-QuIC	BH	YES	DLB	7/2	Brain samples with diffuse neocortical DLB showed 50% seeding dose (SD50) values of 107~1010/g. An estimated SD50 value of ~105/g brain was found for limbic DLB. DLB was distinguished from other neurological and neurodegenerative illnesses by the RT-QUIC assay. In addition. despite the Ser129 phosphorylation state, reactions seeded with oligomer-like species surprisingly recreated the prion-like seeding, but not with insoluble aggregates of r-α-syn.
Groveman et al., 2018 [17]	RT-QuIC	CSF	NO	PD DLB	12/31 17/31	Achieved 93% diagnostic sensitivity and 100% specificity. The quantification of α-syn D seeding activity in CSF samples was made possible using end-point dilution analyses, which also enabled for detection in as little as 0.2 μL.
Kang et al., 2019 [24]	RT-QuIC PMCA	CSF CSF	NO NO	PD PD	105/79 105/79	Clinical parameters, such as the severity or duration of the condition, did not correspond with assay data.
Manne et al., 2019a [20]	RT-QuIC	CSF ΒH	NO YES	PD PD DLB	15/11 11/19 5/19	RT-QuIC: 100% sensitivity and specificity. PD and DLB BH samples: 94% sensitivity and 100% specificity.
Garrido et al., 2019 [27]	RT-QuIC	CSF	NO	LRRK2-PD iPD NMCs of LRRK2	15/10 10/10 16/10	Achieved 90% sensitivity and 80% specificity. No clinical differences were detected between LRRK2-PD patients with positive and negative RT-QuIC.
Van Rumund et al., 2019 [28]	RT-QuIC	CSF	NO *^1^	PD DLB MSA α-synucleinopathy with vasculopathy	53/52 1/52 17/52 11/52	Achieved 84% accuracy, 75% sensitivity, and 94%, specificity α-synucleinopathies from non-α-synucleinopathies and controls.
Bongianni et al., 2019 [36]	RT-QuIC	CSF	YES	DLB MSA LBD/AD LBD/PART LBD/CJD	7/49 1/49 15/49 2/49 3/49	Achievied 92.9% sensitivity and 95.9% specificity from α-synucleinopathies vs. non-alpha-synucleinopathies.
Han et al., 2020 [21]	RT-QuIC	BH	YES	DLB	7/6	α-syn seeding activity varies between patients with detectable dilutions ranging from 10^−3^ to 10^−8^ dilutions of brain tissue and is stable under exposures to the cycles of freezing, thawing, and sonication.
Rossi et al., 2020 [30]	RT-QuIC	CSF	YES	DLB Mixed LBD *^2^ MSA	14/81 7/81 2/81	Overall 95.3%sensitivity for DLB, PD, iRBD, and PAF. Achieved 98% specificity in a neuropathological cohort of 101 cases without LB pathology.
			NO	DLB PD iRBD PAF MSA	34/62 71/62 18/62 28/62 31/62	
Shahnawaz et al., 2020 [22]	PMCA	CSF	NO	PD MSA	94/56 75/56	Achieved 95.4% overall sensitivity PD vs. MSA, and
Orrú et al., 2020 [33]	RT-QuIC	CSF	NO	PD	108/85	95% of the areas under the curve were found in receiver operating characteristic analyses comparing the diagnosis of patients to healthy controls. The reaction times required to achieve 50% maximum fluorescence were linked with the scores on the REM sleep behavioral disorder questionnaire.
Singer et al., 2020 [23]	PMCA	CSF	NO	PD MSA DLB	16/29 62/29 13/29	NFL and α-syn oligomers in CSF faithfully differentiate early MSA not only from controls but also from LB synucleinopathies.
Concha-Marambio et al., 2021 [9]	PMCA	CSF	NO	PD SWEDD	30/30 20/30	Achieved 96.2% sensitivity 96.7% specificity for PD vs. controls at baseline. Achieved 96.4% sensitivity and 93.8% specificity at 4 years follow-up.
Iranzo et al., 2021 [38]	RT-QuIC	CSF	NO	iRBD	52/40	Achieved 90% sensitivity and specificity with increased risk of subsequent PD or DLB diagnosis.
Bargar et al., 2021 [35]	RT-QuIC	CSF	YES	PD DLB	88/68 58/68	Achieved 98% sensitivity and 100/5 specificity.
Brockmann et al., 2021 [26]	RT-QuIC	CSF	NO	sporadic PD PD GBA PD LRRK2 PD recessive *^3^ sporadic DLB DLB GBA NMCs *^4^	107/26 99/26 9/26 20/26 33/26 16/26 14/26	According to RT-QuIC kinetic characteristics, patients with severe GBA mutations had the highest seeding activity among PD patients and the largest percentage of samples with four out of four positive replicates. Among DLB patients, 79% of those with wildtype DLB exhibited positive α-syn seeding, while 100% of those with GBA mutations did. Decreased CSF levels of proteins were associated with α-syn proteostasis and α-syn seeding activity.
Poggiolini et al., 2021 [29]	RT-QuIC	CSF	ΝO *^5^	PD MSA iRBD	74/55 24/55 45/55	Achieved 64% sensitivity in iRBD 90% sensitivity in 3/4 longitudinal iRBD 9 out of 14 convertors to synucleionopathy with baseline RT-QuIC positive.
Compta et al., 2022 [34]	RT-QuIC	CSF	ΝO	PD MSA Taupathies (23 PSP, 12CBD)	20/19 37/19	Achieved 9% sensitivity with diagnostiv revision of MSA, 100% specificities against controls, 91% against taupathies.
Hall et al., 2022 [32]	RT-QuIC	CSF	NO	PD PDD MSA Controls converted to LBD	50/47 14/47 15/47 2/47	Standard LBD vs. no LB pathology: 100% sensitivity and 93% specificity. LB pathology in the cortex vs. cases with no LBs or LBs present only in the olfactory bulb: 97% sensitivity and 93% specificity
			YES	standard LBD *^6^ non-standard LBD *^7^	25/53 23/53	
Garrido et al., 2022 [27]	RT-QuIC	BH SN	YES	LRRK2-PD LTP+ LTP+ controls	3/7 *^8^ 7/7	Achieved 100% accuracy RT-QuIC in SN and CSF LRRK2-PD samples.
		BH AC	YES	LRRK2-PD LTP+ LTP+ controls	3/8 7/8	Achieved 100% sensitivity inSN iPD.
		CSF	YES	LRRK2-PD LTP+ LTP+ controls	2/6 6/6	Negative in the SN control brains.
Siderowf et al., 2023 [14]	RT-QuIC	CSF	NO	PD SWEDD NMCs of GBA NMCs of LRRK2 Prodromal patients	545/163 54/163 151/163 159/163 51/163	Achieved 87·7% sensitivity for PD 98·6% sensitivity for PD + typical olfactory deficit 96.3% specificity for healthy controls 98·6%.
Concha-Marambio et al., 2023 [25]	PMCA	CSF	NO	PD DLB MSA iRBD vascular PD	95/64 2/64 2/64 29/64 3/64	Achieved 98% accuracy in baseline PD-CSF. Results for α-syn-SAA were more in concordance with the final diagnosis than the initial one, since 14 patients were reclassified as not having α-syn aggregation disease. When it came to synucleinopathies, α-syn-SAA and DAT-SPECT had better agreement with the final diagnosis.

*^1^ Only 2% of the cases are autopsy-confirmed. *^2^ Mixed LBD includes CJD with DLB (*n* = 2), CJD with brainstem LBD (*n* = 3), and other primary diagnoses with limbic LBD (*n* = 1) or brainstem LBD (*n* = 1). *^3^ Recessive PD includes patients with mutations in parkin, PINK-1 or DJ-1. *^4^ Νon-manifesting carriers include Carriers of GBA (*n* = 10), LRRK2 (*n* = 3) or recessive (*n* = 1). *^5^ 32 out of 55 controls were autopsy samples. *^6^ standard LBD included cases with PD, PD with AD and DLB. *^7^ non-standard LBD includes AD with Lewy bodies not meeting criteria for DLB or PD, and incidental LBD. *^8^ Controls include LRRK2-PD without LTP and LTP-controls. Abbreviations: AC= anterior cingulate gyrus, SN= substantia nigra, LTP= Lewy Type pathology, NMCs = non-manifesting carriers of mutations in genes related to LBD.

**Table 2:** Studies on α-syn-SAAs using peripheral tissue samples.

Author	Tissue Type	Assay Type	Autopsy	LBD (Cases/Controls)	Main Outcomes
De Luca et al., 2019 [39]	OΜ	RT-QuIC	NO	PD (18/18) MSA (11/18)	High efficiency of α-syn aggregates in MSA and PD olfactory mucosa with different structural/ biochemical features
Stefani et al., 2021 [40]	OΜ	RT-QuIC	NO	PD (41/59) iRBD (63/59)	The specificity was high (89.8%), while the sensitivity for isolated RBD with PD was lower than controls (45.2%), with 78.6% of i RBD patients with positive α-syn. RT-QuIC and 21.4% with negative α-syn RT-QuIC (*p*< 0.001) reported olfactory impairment. When isolated RBD patients tested positive for olfactory mucosa α-syn RT-QuIC, the degree of olfactory impairment was greater than in those who tested negative.
Perra et al., 2021 [41]	OΜ	RT-QuIC	NO	probable DLB (32/38) prodromal DLB (5/38) DLB/AD (6/38)	For olfactory mucosa and CSF, the accuracy of real-time quaking-induced conversion assay results and clinical diagnoses was 86.4% and 93.8%, respectively.
	CSF	RT-QuIC	NO	probable DLB (10/32) DLB/AD (6/32)	
Bargar et al., 2021 [35]	OM	RT-QuIC	NO	PD (13/11) MSA-P (20/11) MSA-C (10/11)	A 96% IAR was achieved between laboratories using the α-syn_RT-QuIC analysis (Kappa = 0.93, 95% CI 0.83–1.00). Specifically, 9/13 patients with PD (sensitivity 69%, IAR 100%) and 18/20 patients with MSA-P (sensitivity 90%, IAR 100%) have α-syn_RT-QuIC seeding activity in their OM. Remarkably, save from one participant in the USA-lab, materials obtained from MSA-C patients did not cause α-syn_RT-QuIC seeding activity. As a result, MSA-P and MSA-C had opposing effects.
Manne et al., 2020 [42]	Frozen SKIN	RT-QuIC	YES	PD (25/25)	Frozen skin tissues: 96% sensitivity and 96% specificity.
	FFPE SKIN	RT-QuIC	YES	PD (12/12)	FFPE SKIN:75% sensitivity and 83% specificity.
Wang et al., 2021 [43]	Abdominal SKIN	RT-QuIC	YES	PD (47/43) LBD (7/43) MSA3/43)	PD: 82% sensitivity and 96% specificity.
		PMCA	YES	Synucleinopathies *^2^ (32/8)	Achieved 93% sensitivity and 93% specificity.
	Scalp SKIN	RT-QuIC	YES	PD (20/10)	The sensitivity and specificity of RT-QuIC were 95% and 100% for posterior cervical and
	Biopsy SKIN *^3^	RT-QuIC	NO	PD (20/21)	leg skin biopsy tissues fromPD and controls without PD, respectively,
		PMCA	NO	PD (10/10)	sensitivity 80% and specificity 90% for PMCA.
Donadio et al., 2021 [44]	SKIN *^4^	RT-QuIC	NO	PD (6/18) MSA (5/18) LBD (4/18) PAF (3/18)	When separating synucleinopathies from non-synucleinopathies and controls, both immunofluorescence and RT-QuIC had high sensitivity and specificity; however, immunofluorescence demonstrated a higher diagnostic accuracy.
	CSF	RT-QuIC	NO	PD (2/13) MSA (2/13) LBD (2/13) PAF (2/13)	
Mammana et al., 2021 [45]	SKIN cervical SKIN thigh CSF SKIN cervical SKIN thigh SKIN leg CSF	RT-QuIC RT-QuIC RT-QuIC RT-QuIC RT-QuIC RT-QuIC RT-QuIC	YES YES YES NO NO NO NO	PD (1/40) iLBD (7/40) DLB (1/40) PD (1/39) iLBD (6/49) DLB (1/39) iLBD (4/30) DLB (1/30) DLB (4/15) PD (4/15) DLB (7/11) PD (4/11) DLB (4/15 PD (5/15) DLB (11/27) PD (7/27)	A 94.1% accuracy (sensitivity, 89.2%; specificity, 96.3%) was achieved in differentiating LBD patients using the skin α-syn real-time quaking-induced conversion assay. In the 17 LBD patients examined in the cervical region, the assay’s sensitivity was 94.1%. The two real-time quaking-induced conversion assay procedures produced comparable diagnostic accuracy (skin, 97.5%; CSF, 98.7%) in patients with both skin and CSF samples.
Kuzkina et al., 2021 [46]	SKIN *^5^	RT-QuIC	NO	PD (34/30)	High degree of inter-rater agreement between the two laboratories (92.2%) and PD diagnostic accuracy of 88.9%. Patients with more advanced illness stages and longer disease duration had higher α-syn seeding activity in RT-QuIC, which was linked with constipation, cognitive impairment, and REM sleep behavior disorder.
Kuzkina et al., 2023 [47]	SKIN *^6^	RT-QuIC	NO	iRBD (38/23) PD (39/23)	A total of 92.2% of PD patients (70% of PD biopsies), 13.3% of controls (7.9% of control biopsies), and 97.4% of iRBD patients (78.4% of iRBD biopsies) all showed evidence of α-synuclein aggregation, with iRBD showing higher seeding activity than PD. Comparing immunohistochemistry to RT-QuIC, the latter was less specific but more sensitive.
Fenyi et al., 2019 [48]	GI rectum	PMCA	ΝO	PD (4/4)	All of the control samples, with the exception of one patient, did not cause α-syn aggregation in the PMCA reaction. In 10 of the 18 cases, GI biopsies from PD patients (2 from the antrum, 1 from the rectum, and 7 from the sigmoid colon) sparked an α-syn aggregation. The PMCA and immunohistochemistry results agreed well since, with the exception of two instances, all PD patients who tested positive for PMCA also tested positive for PASH.
	GI sigmoid	PMCA	NO	PD (12/7)	
	GI antrum	PMCA	NO	PD2/-	
Manne et al., 2019 [20]	SMG	RT-QuIC	YES	PD (13/16) iLBD (3/16)	When compared to control tissues, the enhanced levels of pathogenic α-syn seeding activity in PD and incidental LBD tissues exhibited 100% agreement. The submandibular gland exhibited a broad dynamic range of pathogenic α-syn seeding activity, according to end-point dilution kinetic studies.
Chahine et al., 2023 [10]	SMG	RT-QuIC	NO	PD (41/14)	Achievied 73.2% sensitivity and 78.6% specificity.
	CSF	PMCA	NO	PD (54/21)	Achievied 92.6% sensitivity and 90.5%specificity.
Luan et al., 2022 [49]	SALIVA	RT-QuIC	NO	PD (75/36) MSA (18/36)	PD: 76.0% sensitivity and 94.4% specificity MSA: 61.1% sensitivity. Patients with PD and MSA did not exhibit any significant changes in the diameter of salivary α-syn fibrils analyzed by electron microscopy or in the thioflavin T fluorescence intensity of salivary α-syn fibrils detected by RT-QuIC test. Notably, patients with PD had a notably shorter lag phase in the RT-QuIC assay than patients with MSA. This finding may have practical applications in differentiating between PD and MSA.
Okuzumi et al., 2023 [50]	SERUM	IP/RT-QuIC	NO	PD (221/128) MSA (39/128) DLB (10/128 RBD (9/128) Parkin-PD (17/128)	When comparing PD and MSA to controls, IP/RT-QuIC demonstrated excellent diagnostic performance, 96%. In a blinded external cohort, IP/RT-QuIC also shown strong diagnostic efficacy in separating patients with PD (86%) and MSA (80%) from controls.

Each sample was analyzed by two different labs. The results for PD and MSA-P subjects showed an interrupter agreement of 100% between the two labs. Among the MSA-C patients, one was positive at USA-lab (10% sensitivity) and none was positive at ITA-lab (0% sensitivity) and among healthy controls, specificities of 91% and 100% were reached at USA-lab and ITA-lab, respectively. *^2^ Synucleinopathies include PD cadavers (*n* = 24), LBD cadavers (*n* = 5) and MSA cadavers (*n* = 3). *^3^ Biopsy skin samples were obtained from the leg or the posterior cervical region. *^4^ Biopsy skin samples were obtained from C7, thigh and leg. *^5^ Biopsy skin samples were obtained from C7, Th10, Thigh and lower leg. *^6^ Biopsy skin samples were obtained from the leg, C7 or Th10. Abbreviations: FFPE Formalin-fixed paraffin-embedded, IP/RT-QuIC immunoprecipitation-based real-time quaking-induced conversion.

## Data Availability

No new data were created or analyzed in this study.

## Supplementary Materials

The following supporting information can be downloaded at https://www.mdpi.com/article/10.3390/ijms251910783/s1.
