# Supplementary material for: Seeding Aggregation Assays in Lewy Bodies Disorders: A Narrative State-of-the-Art Review
Source: Int J Mol Sci. 2024 Oct 7;25(19):10783. doi: 10.3390/ijms251910783 (PMC11477186; doi:10.3390/ijms251910783)
Supplement: Supplementary file 1 [file ijms-25-10783-s001.zip › ijms-3237789-supplementary.pdf]

Supplementary Material 1

The typically soluble prion protein (substrate) changes into an amyloid fibril rich in beta-sheet if PrPSc is present in the test sample. It was previously a highly alpha-helical structure. Samples are cultivated in a buffer solution containing the substrate (protein monomer) at a predetermined temperature. Sample aggregates that have already been created act as templates, polymerizing at their extremities at the expense of the substrate. During the shaking/sonication stage, the developed aggregates break into smaller pieces and create more polymerization sites. The incubation and fragmentation processes are carried out repeatedly in a cyclic manner to cause an exponential expansion of the pathogenic aggregates [1](figure 1)

Amyloid fibrils generally undergo a seeding aggregation process that can be broken down into three phases: a) a LAG phase, during which the substrate interacts with the preformed fibriland until the fluorescence reaches a predetermined threshold for positivity; b) a growth phase, during which the fibrils elongate and produce new seeding surfaces through fragmentation or secondary nucleation; and c) a plateau phase, signifying that nearly all of the available substrate has been used [12,13]. Typically, the aggregation kinetic is enhanced even at low concentrations of PRPsc[12] (Figure 1)~~.~~

This assay employs a recombinant PrPc, which may contain amino acid sequences from several species, as a reaction substrate. The aggregation process can be observed in real time thanks to the use of ThT dye. The samples are incubated at a high >42^0 C temperature in the buffer solution containing the substrate. Every sample is analyzed in four copies using a multi-well plate. When the PrPsc protein is present in the sample, shaking the sample intermittently causes the amyloid fibrils to break into smaller pieces after the sample has been incubated in the buffer solution. To turn additional substrate into amyloid fibrils, these steps are repeated multiple times. When the sample contains PrPsc, the fluorescent ThT dye creates a kinetic curve showing the LAG, growth, and plateau phases of the typical aggregation process against time. A sample is considered positive if it exhibits a seeding effect in at least two of the four repetitions. The final products of the RT-QuIC assay exhibit limited resistance to PK digestion.
